# Altered effective connectivity of posterior thalamus in migraine with cutaneous allodynia: a resting-state fMRI study with granger causality analysis

**DOI:** 10.1186/s10194-016-0610-4

**Published:** 2016-02-27

**Authors:** Ting Wang, Ning Chen, Wang Zhan, Jia Liu, Junpeng Zhang, Qi Liu, Hua Huang, Li He, Junran Zhang, Qiyong Gong

**Affiliations:** Department of Medical Information Engineering, School of Electrical Engineering and Information, Sichuan University, No.24, South Section One, First Ring Road, Chengdu, 610065 P.R China; Huaxi MR Research Center (HMRRC), Department of Radiology, West China Hospital of Sichuan University, Chengdu, P.R China; Department of Neurology, West China Hospital of Sichuan University, Chengdu, P.R China; Neuroimaging Center, University of Maryland, College Park, MD USA

**Keywords:** Migraine, Cutaneous allodynia, Resting-state functional magnetic resonance imaging, Granger causality analysis, Effective connectivity, Posterior thalamus

## Abstract

**Background:**

Most migraineurs develop cutaneous allodynia (CA) during migraine, and the underlying mechanism of CA in migraine is thought to be sensitization of the third-order trigeminovascular neurons in the posterior thalamic nuclei. This study aimed to investigate whether the ascending/descending pathway associated with the thalamus is disturbed in migraineurs with CA (MWCA) using effective connectivity analysis of resting-state functional magnetic resonance imaging.

**Methods:**

Thirty four migraineurs without aura (14 MWCA and 20 migraineurs without CA (MWoCA)) and 25 matched healthy controls (HC) were recruited in the study. The effective connectivity pathways associated with the posterior thalamus (PTH) were investigated using the Granger causality analysis. We chose bilateral PTH as two individual seeds, and compared MWCA with MWoCA and HC, respectively. Spearman correlation analysis was performed to test the correlation between the abnormal effective connectivity and the allodynia severity of MWCA.

**Results:**

Compared with MWoCA, MWCA showed decreased inflows from the left limbic regions and dorsal medial prefrontal cortex (dmPFC) to the ipsilateral PTH, as well as increased inflow from the right ventral medial prefrontal cortex (vmPFC) to the ipsilateral PTH; no significantly different outflows from the bilateral PTH to other regions were found. Compared with HC, MWCA showed increased outflows from the left PTH to the bilateral vmPFC, decreased outflows from the right PTH to the bilateral temporoparietal areas, decreased inflow from the left parietooccipital area to the ipsilateral PTH, and increased inflows from the right dorsolateral prefrontal cortex and the bilateral temporoparietal areas to the right PTH. Correlation analyses revealed that the disturbed connectivities between PTH and cuneus, as well as PTH and middle frontal gyrus were associated with the allodynia severity of MWCA.

**Conclusions:**

MWCA demonstrated disrupted effective connection pathways between the PTH and other cortical or subcortical regions that participated in multi-dimentional pain processing. Our findings highlight the dysfunctional ascending and descending pain network at the thalamic-level and may help to illuminate the possible pathophysiologic mechanisms of CA.

## Background

Migraine is a disabling and common neurological disorder, typically characterized by unilateral, throbbing, pulsating headache [1]. It affects about 12 % of general population and causes substantial personal and social burden [2]. During headache attacks, more than 60 % of migraineurs develop cutaneous allodynia (CA) [3, 4], which is defined as a painful perception of an innocuous stimulation of the skin [5]. CA is associated with high frequency of attacks, and has been proposed as a risk factor for progression to chronic migraine [6, 7]. In addition, it has been also proposed as a predictor of poor response to triptan therapy [8]. Therefore, probing the mechanism of CA in migraine may have significant implications for our understanding of the pathophysiology of migraine and its prognosis.

The hypothetical theory regarding the pathophysiological mechanism of CA is central sensitization of trigeminal neurons that take inputs from the intracranial and extracranial structures [9–11]. Central sensitization is a condition in which neurons have lower activation thresholds, increased responsiveness to afferent inputs, increased spontaneous activity, and enlarged receptive fields [12]. It is hypothesized that sensitization of the second-order trigeminovascular neurons in the spinal trigeminal nucleus propels the development of cephalic allodynia, and sensitization of the third-order trigeminovascular neurons in the posterior thalamic nuclei mediates the development of extracephalic allodynia [13, 14]. Functional neuroimaging studies in humans and animals have shown activation of the trigeminal neurons in the posterior/dorsal thalamic nuclei in migraine [9, 15, 16]. In addition, activation of the trigeminal system has been found to be associated with the ascending nociceptive transmission via the trigemino-thalamo-cortical pathway [17]. As we know that thalamus is a relay region subserving both sensory and motor processing, with axonal fibers not only projecting out to the cerebral cortex in all directions but also receiving feedback informations from those multiple cortical areas [18]. For pain perception, thalamus is believed to modulate ascending nociceptive information before transmitting nociceptive inputs to cortical structures [19, 20]. Furthermore, thalamus is also a part of the descending pain inhibition pathways that modulate nociceptive inputs from spinal trigeminal nucleus [21]. However, how the posterior thalamus affects the brain systems and whether it is affected by those brain regions in migraineurs with CA are poorly understood.

Effective connectivity and functional connectivity are two effective techniques to address the integration of functionally specialized areas in human brain [22]. They both allow the inference of regarding communication between spatially remote brain regions, whereas effective connectivity, utilizing information about time-lagged relationships between brain regions, allows additional inferring about the directionality of information transfer within functionally connected networks [23]. Previous resting-state functional magnetic resonance imaging (rs-fMRI) studies of migraine have found abnormal functional connectivity between thalamus and other pain matrix regions, such as periaqueductal grey (PAG), nucleus cuneiformis (NCF), or the affective regions (including insula, anterior cingulate cortex, and amygdala) [24–26]. However, these studies are neither directly investigating the CA in migraine, nor are applying the thalamic-level effective connectivity analysis. Therefore, we hypothesize that studying the direct influence (i.e., effective connectivity) of thalamus with other structures can strengthen our understanding of the neural circuitry of CA in migraine.

In the present study, we aim to investigate the effective connectivity patterns of bilateral posterior thalamus with the rest of the brain in migraineurs with CA. Granger causality analysis (GCA) is a special approach to explore such effective connectivity among regions, and can be used to obtain the deductive network based on certain hypothetical seed region without requiring prior knowledge [27]. So by employing the GCA method on rs-fMRI data, we can reconstruct an effective connectivity network associated with thalamus, which would provide us with fresh insights into the descending or ascending pain pathways at the thalamic-level in migraine with CA. To the best of our knowledge, this is the first study to examine the causal interactions between the posterior thalamus and the rest brain regions in migraineurs with CA using rs-fMRI.

## Methods

### Subjects

Thirty-four (*N* = 34) right-handed migraineurs (aged 19–44 years) without aura as well as 25 age-, sex-, and handness-matched healthy controls were recruited for this study. The diagnosis of migraine without aura was made according to the Second Edition of the International Classification of Headache Disorder (ICHD- II) [28]. The protocol of this study was approved by the Medical Ethics Committee of West China Hospital, Sichuan University, and all participants provided written informed consent. To avoid any possible pharmacological interference, all subjects had to be weaned off analgesic drugs 1 week or longer, not in preventive treatment and had not used any other drugs for at least 1 month prior to study participation. Subjects did not having a migraine attack at least 72 h prior to scanning. In addition, patients would be excluded if they had a migraine precipitated during the 2-day following-up. Potential subjects were excluded if they had any contraindication to MRI, had a prior brain injury, had a neurologic disorder other than migraine, had a psychiatric disorder other than anxiety or depression, or if they had any acute or chronic pain disorder other than migraine.

### Clinical parameters

The Allodynia Symptom Checklist-12 (ASC-12) scale was administrated to divide all enrolled migraineurs into two groups: migraineurs with or without cutaneous allodynia (MWCA or MWoCA). ASC-12 is a 12-item questionnaire presented interictally to migraine patients, estimating the prevalence and severity of CA in the migraine population. The yielding score of each subject was placed into one of 4 categories: 0–2 = no allodynia; 3–5 = mild allodynia; 6–8 = moderate allodynia; 9 or more = severe allodynia. Then all participants were interviewed regarding demographic features (e.g., age, sex, and education). Additional information including the migraine history (e.g., onset age, frequency and duration of attacks, headache characteristic, and the pain intensity (evaluated with the visual analogue scale (VAS))) and the impact of headache (evaluated with the Migraine Disability Assessment Scale (MIDAS) [29] and Headache Impact Test (HIT)-6 [30]) was collected in those ones with migraine. For all subjects, psychiatric assessment, including 24-Hamilton Depression Scale (24- HAMD) [31] and 14-Hamilton Anxiety Scale (14-HAMA) [32] was also administered to assess the depression and anxiety state.

### Statistical analysis of clinical data

Demographic and clinical features of MWCA compared to MWoCA and HC respectively were conducted using an independent sample *t*-test or chi square test, as appropriate.

### Data acquisition

The experiment was performed on a 3.0 Tesla scanner (Trio Tim, Siemens, Erlangen, Germany) with a 16-channel birdcage head coil, and tightly padded clamps were used to minimize head motion. A routine T1 weighted imaging was first performed, and then the resting-state fMRIs were obtained by using an echo-planar imaging sequence with the following parameters: voxel size 3.75 × 3.75 × 5 mm^3^, TR 2000 ms, TE 30 ms, FOV 240 × 240 mm^2^, matrix 64 × 64, and slice thickness 5 mm with no gap, number of slices 30, number of time points 180. Throughout the scanning, subjects were instructed to lay in the scanner supine, relax, close their eyes but be awake.

### Data spatial processing

The data were pre-processed using SPM8 (The Wellcome Department of Cognitive Neurology, London, UK, www.fil.ion.ucl.ac.uk/spm/software/spm8). The first five time points of the resting-state data were discarded due to instability of the initial MRI signal, leaving 175 time points remaining for further processing. Then the functional images were slice-timing corrected and realigned to the first volume using a six-parameter rigid body transformation. Subjects with maximum head translation exceeded 2 mm or maximum rotation exceeded 2° were excluded. The mean image generated was then spatially normalized into standard stereotactic space, using the Montreal Neurological Institute (MNI) echo planar imaging (EPI) template. Computed transformation parameters were applied to all functional images, interpolated to isotropic voxels of 2 mm^3^ and the resulting images were smoothed using an 4-mm full-width half-maximum (FWHM), isotropic Gaussian kernel. Then, using the Data Processing Assistant for resting-state fMRI (DPARSF) package [33], linear drift was removed. A band-pass frequency filter (0.01 < f < 0.08 Hz) was then applied to reduce physiological high frequency noise [34]. To further reduce the effects of confounding factors unlikely to be involved in specific regional correlation, we also removed several sources of spurious variance by linear regression, including six head motion parameters, and average signals from cerebrospinal fluid, white matter according to previous fMRI studies [35, 36].

### Granger causality analysis and statistical analysis

We used Granger causality to describe the effective connectivity between the reference time series of the seed regions (left and right posterior thalamus (PTH)) and the time series of each voxel within the whole brain. The coordinates (peak MNI coordinates: the right PTH = 3, −14, 6; the left PTH = −6, −21, −3) were obtained based on previous studies that showed structural or functional alterations in PTH in migraineurs [37, 38]. Two 6 mm-radius sphere seeds based on the peak coordinates were then designed for GCA. Bivariate first-order coefficient-based voxelwise GCA was performed using the REST-GCA in the REST toolbox (http://www.restfmri.net) [39]. Granger causality estimates the causal effect of the seed region on every other voxel in the brain (X to Y effect), as well as the Y to X effect, the causal effect of every voxel in the brain (Y) on the seed region (X). A positive coefficient from X to Y indicates that activity in region X exerts a causal influence on the activity region Y in the same direction (i.e., positive influence). Similarly, a negative coefficient from X to Y suggests that the activity of region X exerts an opposing directional influence on the activity of region Y (i.e., negative influence). Using this approach, we are able to build a Granger-causal model based on the temporal elements of regional BOLD activity. The generated voxel-wise GCA maps were than transformed to z scores to improve the normality [40]. Two-tailed two-sample *t*-tests were conducted on the causal effects to compare MWCA to MWoCA and HC respectively with a Gaussian random field (GRF)-corrected significance (voxel-level of *P* < 0.01 and joint cluster-level of *P* < 0.05). Age, sex ratio, and education were applied as covariates in the two-sample *t*- tests.

### Clinical correlation analysis

To investigate the association between allodynia severity and abnormal effective connectivity, the regions showing significantly different (increased or decreased) Granger influences between the MWCA and MWoCA, or MWCA and HC comparisons were extracted as regions of interest (ROIs). Mean Granger causality values within these ROIs were correlated against the ASC scores of MWCA using Spearman correlation analysis. Statistical analyses were performed in SPSS 17.0 (SPSS Inc., Chicago, Illinois, USA) and threshold was set at *P* < 0.05.

## Results

### Demographics and clinical data

Demographic and psychiatric characteristics of all subjects were given in Table 1. No subject was excluded due to head movement. Of the 34 migraineurs without aura, 14 were classified as MWCA and 20 as MWoCA according to their ASC scores. All the enrolled MWCA were with mild (3–5 scores) or moderate (6–8 scores) allodynia [4]. All subjects were right-handed. No significant difference was found for gender, age, or education either between MWCA and MWoCA, or MWCA and HC groups. Both the mean 24-HAMD and 14-HAMA scores of MWCA were significantly higher than those of HC (*P* value was 0.000 and 0.004, respectively), while they were not significantly different between the two migraine groups.

**Table 1:** Demographic and psychiatric characteristics of the study participants

Information	MWCA (*n* = 14)	MWoCA (*n* = 20)	HC (*n* = 25)	*P* value in MWCA vs. MWoCA	*P* value in MWCA vs. HC
Male/female	1/13	6/14	10/15	0.787	0.649
Age (mean ± SD, year)	28.93 ± 7.90	28.10 ± 8.50	28.60 ± 6.68	0.751	0.415
Education (mean ± SD, year)	15.36 ± 2.50	14.75 ± 3.64	15.76 ± 2.49	0.208	0.754
24-HAMD (mean ± SD)	8.64 ± 5.53*	6.05 ± 6.53*	2.92 ± 1.96	0.910	0.000
14-HAMA (mean ± SD)	6.71 ± 6.52*	4.25 ± 5.62*	2.20 ± 1.29	0.751	0.004

*24-HAMD* 24-item Hamilton depression scale, *14-HAMA* 14-item Hamilton anxiety scale, *HC* healthy control, *MWCA* migraineurs with cutaneous allodynia, *MWoCA* migraineurs without cutaneous allodynia, *SD* standard deviation

*P < 0.05, comparing to the control group; No significant difference was found between the two migraine groups

Table 2 showed the headache features of the two migraine groups. In respect to the ASC-12, the MWCA group got a significantly higher score comparing to the MWoCA group (*P* = 0.012). There were no significant difference between the two groups in course of migraine disease and average headache intensity assessed by VAS. HIT-6 and MIDAS also achieved balance between the two migraine groups. However, MWoCA showed a significantly higher headache frequency and attack duration than MWCA (*P* = 0.018 and 0.028, respectively).

**Table 2:** Headache features of the patients with migraine

Information	MWCA (*n* = 14)	MWoCA (*n* = 20)	*P* value
Years with Migraine (mean ± SD, year)	8.43 ± 7.20	9.6 ± 6.42	0.914
Headache Frequency (mean ± SD, attacks/month)	3.50 ± 1.91*	6.20 ± 7.16	0.018
Attack Duration (mean ± SD, hour)	13.64 ± 10.84*	16.48 ± 22.16	0.028
ASC-12 (mean ± SD)	5.14 ± 1.56*	0.65 ± 0.88	0.012
VAS (mean ± SD)	61.79 ± 16.36	54.75 ± 10.94	0.120
HIT-6 (mean ± SD)	56.07 ± 16.38	60.50 ± 5.84	0.256
MIDAS (mean ± SD)	15.00 ± 11.69	16.65 ± 19.53	0.241

*ASC-12* 12-item Allodynia symptom checklist, *HIT-6* six-item headache impact test, *MIDAS* migraine disability assessment, *MWCA* migraineurs with cutaneous allodynia, *MWoCA* migraineurs without cutaneous allodynia, *SD* standard deviation, *VAS* visual analogue scale, score range 0–100

*P < 0.05, comparing to the MWoCA group

### Abnormality for causal influence to and from the left PTH

**Fig. 1:**
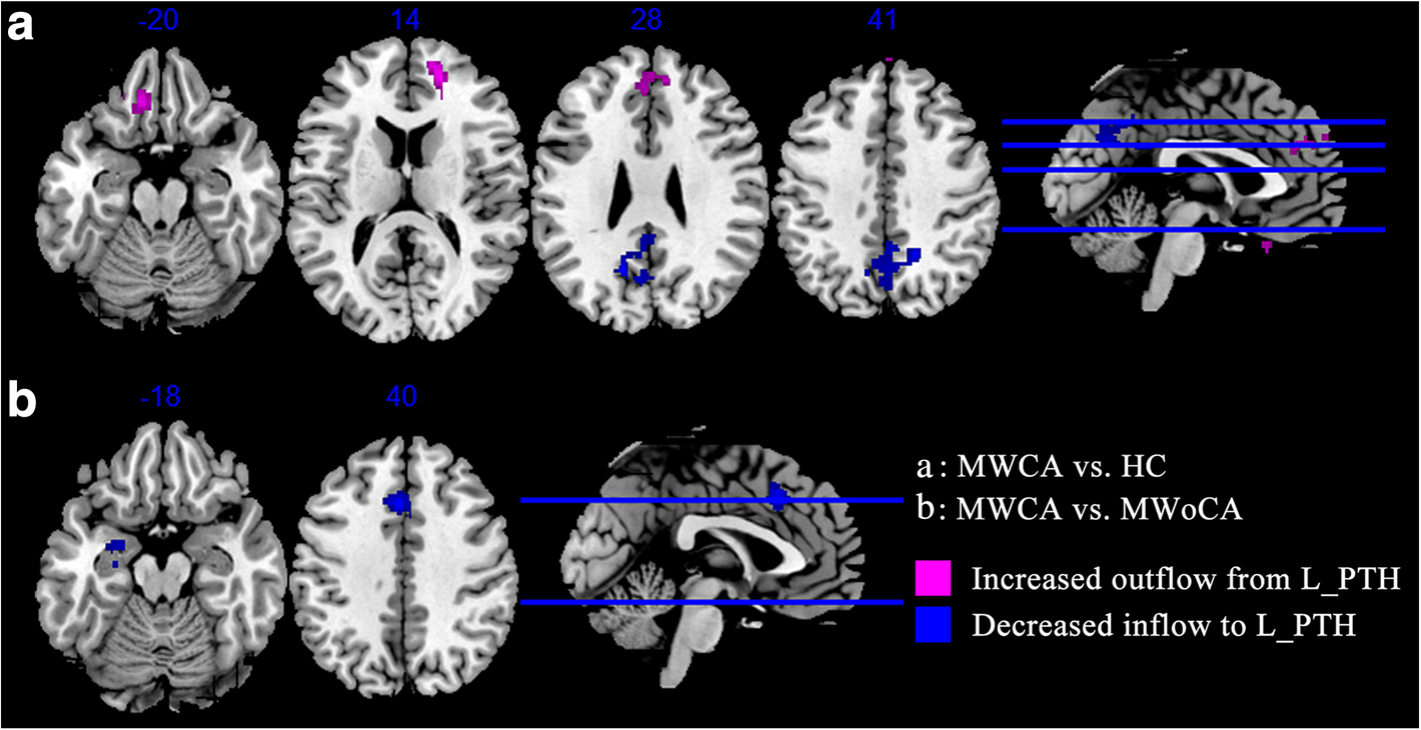
Altered effective connectivity to and from the left PTH in MWCA compared with HC (**a**) and MWoCA (**b**) (voxel-level *P* < 0.01 and cluster-level *P* < 0.05, Gaussian random field corrected) respectively. Compared with HC, MWCA shows decreased inflow from the L_PTH to the left cuneus, precuneus, and PCC, and increased outflow to the left RG, mOFC, SFG, and ACC (**a**). Compared with MWoCA, MWCA shows decreased inflow from ipsilateral amygdala, hippocampus, putamen, pallidum, mSFG, SMA, and MCC (**b**). ACC, anterior cingulate cortex; HC, healthy control; L_PTH, right posterior thalamus; MCC, middle cingulate cortex; mOFC, medial orbitofrontal cortex; mSFG, medial superior frontal gyrus; MWCA, migraineurs with cutaneous allodynia; MWoCA, migraineurs without cutaneous allodynia; PCC, posterior cingulate cortex; PTH, posterior thalamus; RG, rectus gyrus; SFG, superior frontal gyrus; SMA, supplementary motor area

Two-sample *t*-test results of resting-state effective connectivity to and from the left PTH were illustrated in Table 3, Figs. 1, and 3a. Compared to HC, MWCA showed significantly decreased causal inflow to the left PTH from the ipsilateral parietooccipital regions (including the cuneus, posterior cingulate cortex (PCC), and precuneus), and significantly increased causal outflow to the bilateral ventromedial prefrontal cortex (vmPFC, including the left rectus gyrus (RG), medial orbitofrontal cortex (mOFC), bilateral mSFG, and right anterior cingulate cortex (ACC)) (Fig. 1a). Both the limbic regions (including the left amygdala, hippocampus, basal ganglia (BG, including the putamen and pallidum)) and dorsomedial prefrontal cortex (dmPFC, including the left medial superior frontal gyrus (mSFG), supplementary motor area (SMA), and bilateral middle cingulate cortex (MCC)) had decreased influence to the ipsilateral PTH in MWCA comparing to those in MWoCA (Fig. 1b). No significantly different causal outflow from the left PTH to rest of brain was found between two migraine comparisons.

**Table 3:** Two sample *t*-test (voxel-level *P* < 0.01 and cluster-level *P* < 0.05 Gaussian random field corrected) of difference for causal influence to and from the left posterior thalamus

Regions	Peak voxel MNI coordinates (mm)			*T* value	Total voxels	Breakdown (number of voxels)
	x	y	z			
MWCA vs. HC						
Causal outflow from L posterior thalamus to the rest of brain (X to Y)						
L Cerebrum//Medial Frontal Gyrus//Rectus_L	−10	34	−20	4.2681	257	Rectus_L (137)
						Frontal_Sup_Orb_L (68)
						Frontal_Med_Orb_L (52)
R Cerebrum//Medial Frontal Gyrus	20	48	14	4.8552	242	Frontal_Sup_Medial_R (86)
						Frontal_Sup_Medial_L (71)
						Frontal_Sup_R (54)
						Cingulum_Ant_R (31)
Causal inflow to L posterior thalamus from the rest of brain (Y to X)						
L Cerebrum//Occipital Lobe//Precuneus//Cuneus_L	−14	−62	28	−4.7685	404	Precuneus_L (189)
						Precuneus_R (127)
						Cuneus_L (45)
						Cingulum_Post_L (43)
MWCA vs. MWoCA						
Causal inflow to L posterior thalamus from the rest of brain (Y to X)						
L Cerebrum//Parahippocampa Gyrus//Amygdala_L	−24	−4	−18	−3.7253	163	Amygdala_L (65)
						Hippocampus_L (53)
						Putamen_L (28)
						Pallidum_L (17)
L Cerebrum//Cingulate Gyrus//Frontal_Sup_Medial_L	−4	20	40	−4.8582	162	Frontal_Sup_Medial_L (65)
						Supp_Motor_Area_L (50)
						Cingulum_Mid_L (35)
						Cingulum_Mid_R (12)

*HC* healthy control, *L* left, *MNI* Montreal Neurological Institute, *MWCA* migraineurs with cutaneous allodynia, *MWoCA* migraineurs without cutaneous allodynia, *R* right

### Abnormality for causal influence to and from the right PTH

**Fig. 2:**
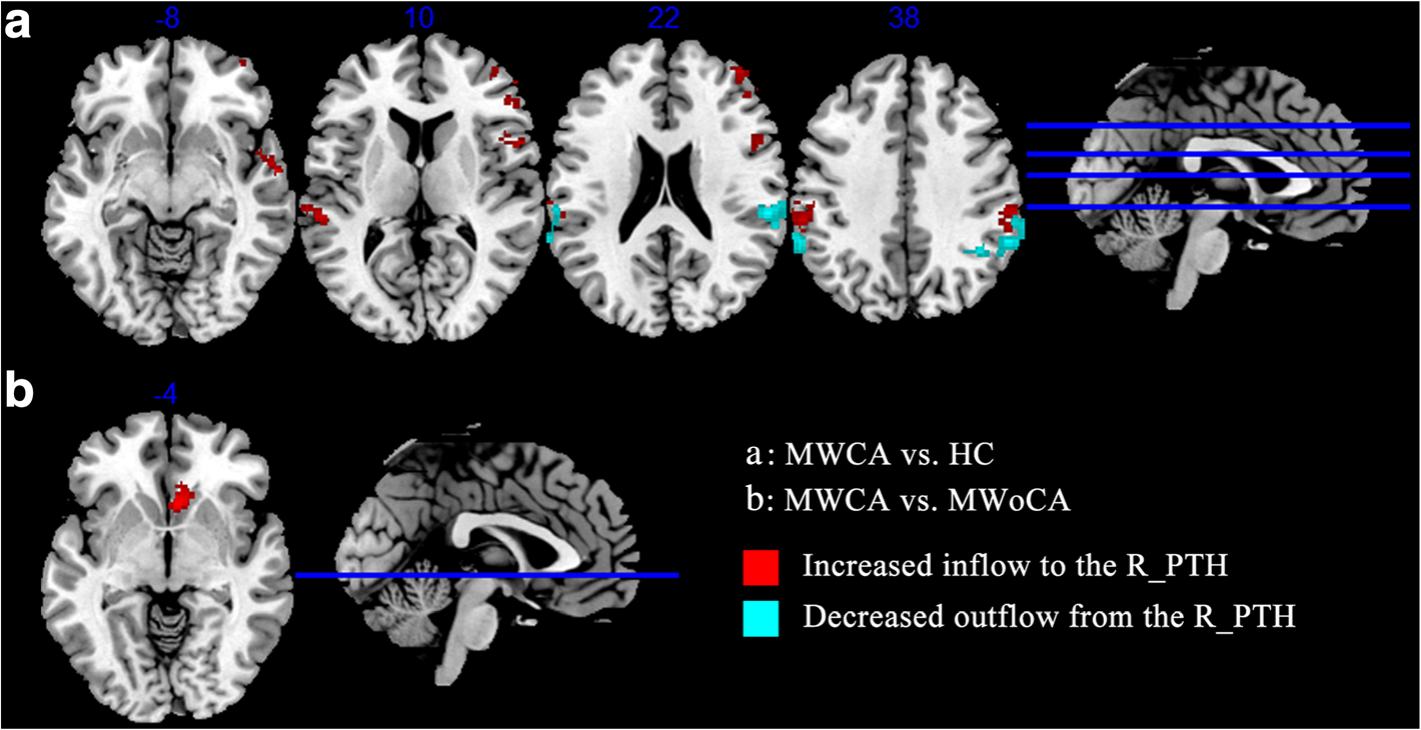
Altered effective connectivity to and from the right PTH in MWCA compared with HC (**a**) and MWoCA (**b**) (voxel-level *P* < 0.01 and cluster-level *P* < 0.05, Gaussian random field corrected) respectively. Compared with HC, MWCA showed increased inflow to R_PTH from the bilateral STG, SMG, IPL, IFG, and MFG, and decreased outflow to the same regions (**a**). Compared with MWoCA, MWCA showed increased inflow from the OC and caudate to the R_PTH (**b**). HC, healthy control; IFG, inferior frontal gyrus; IPL, inferior parietal lobule; MFG, middle frontal gyrus; MWCA, migraineurs with cutaneous allodynia; MWoCA, migraineurs without cutaneous allodynia; OC, olfactory cortex; PTH, posterior thalamus; R_PTH, right posterior thalamus; SMG, supramarginal gyrus; STG, superior temporal gyrus

**Fig. 3:**
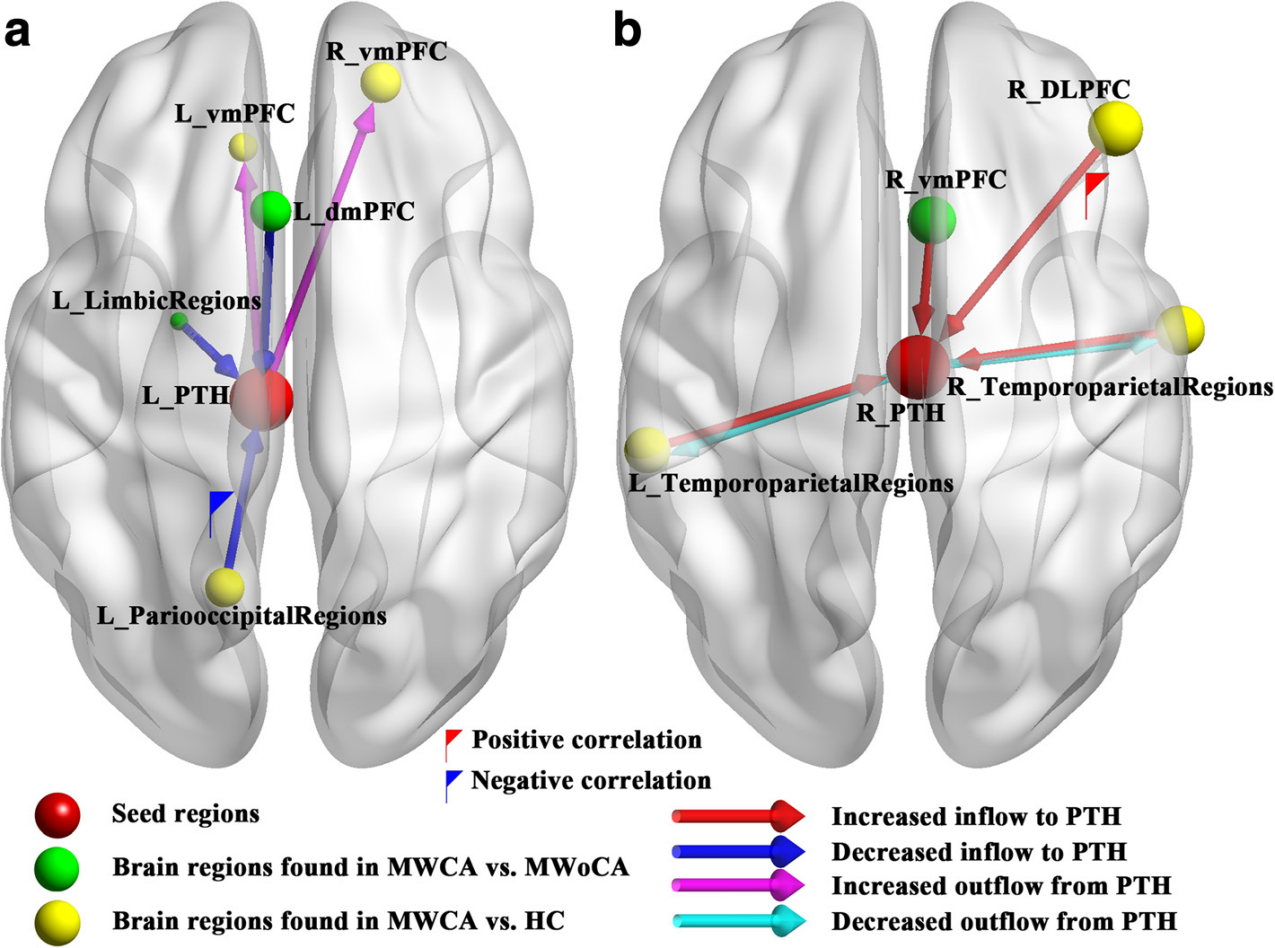
Illustration of the abnormal thalamic-level Granger-causal model in MWCA compared with the two control groups. **a** Abnormal effective connectivity pathways associated with the Left PTH. **b** Abnormal effective connectivity pathways associated with the right PTH. The node size is represented by absolute T-value of corresponding cluster. The red and blue flags represent the positive and negative correlation between the strength of abnormal effective connectivity pathway and ASC (index of allodynia severity), respectively. DLPFC, dorsolateral prefrontal cortex; dmPFC, dorsal medial prefrontal cortex; HC, healthy control; L, left; MWCA, migraineurs with cutaneous allodynia; MWoCA, migraineurs without cutaneous allodynia; PTH, posterior thalamus; R, right; vmPFC, ventral medial prefrontal cortex

As shown in Table 4, Figs. 2a, and 3b, compared with HC, MWCA group demonstrated significantly increased causal inflow to the right PTH from the bilateral temporoparietal areas (including the superior temporal gyrus (STG) and inferior parietal lobule (IPL, including the supramarginal gyrus and angular gyrus)), as well as decreased causal outflow to the bilateral temporoparietal areas; MWCA also exhibited increased causal inflow to the right PTH from the ipsilateral dorsolateral prefrontal cortex (DLPFC, including the opercular, triangular, and orbital part of right inferior frontal gyrus (IFG) and the orbital part of middle frontal gyrus (MFG)). Compared to MWoCA, MWCA had increased inflow to the right PTH from the ipsilateral vmPFC (including the olfactory cortex and caudate nucleus) (Fig. 2b) and no significantly different causal outflow from the right PTH to rest of brain was found.

**Table 4:** Two sample *t*-test (voxel-level *P* < 0.01 and cluster-level *P* < 0.05 Gaussian random field corrected) of difference for causal influence to and from the right posterior thalamus

Regions	Peak voxel MNI coordinates (mm)			*T* value	Total voxels	Breakdown (number of voxels)
	x	y	z			
MWCA vs. HC						
Causal outflow from R posterior thalamus to the rest of brain (X to Y)						
R Cerebrum//Inferior Parietal Lobule//Temporal_Sup_R	58	−30	22	−4.146	473	SupraMarginal_R (346)
						Parietal_Inf_R (63)
						Temporal_Sup_R (52)
						Angular_R (12)
L Cerebrum//Supramarginal Gyrus//Parietal_Inf_L	−60	−46	38	−3.852	198	SupraMarginal_L (156)
						Parietal_Inf_L (27)
						Temporal_Sup_L (15)
Causal inflow to R posterior thalamus from the rest of brain (Y to X)						
R Cerebrum//Middle Temporal Gyrus//Temporal_Sup_R	60	−6	−8	4.509	281	Frontal_Inf_Oper_R (154)
						Temporal_Sup_R (69)
						Temporal_Pole_Sup_R (40)
						Rolandic_Oper_R (18)
R Cerebrum//Frontal_Mid_R	46	38	20	5.0173	490	Frontal_Mid_R (265)
						Frontal_Inf_Tri_R (155)
						Frontal_Mid_Orb_R (59)
						Frontal_Inf_Orb_R (11)
L Cerebrum//Temporal_Sup_L	−56	−32	10	4.1279	318	SupraMarginal_L (172)
						Temporal_Sup_L (65)
						Parietal_Inf_L (45)
						Postcentral_L (36)
R Cerebrum//Inferior Parietal Lobule//SupraMarginal_R	60	−30	38	3.9594	216	SupraMarginal_R (191)
						Parietal_Inf_R (25)
MWCA vs. MWoCA						
Causal inflow to R posterior thalamus from the rest of brain (Y to X)						
R Cerebrum//Anterior Cingulate//Olfactory_R	6	18	−4	4.3433	148	Caudate_R (101)
						Olfactory_R (47)

*HC* healthy control, *L* left, *MNI* Montreal Neurological Institute, *MWCA* migraineurs with cutaneous allodynia, *MWoCA* migraineurs without cutaneous allodynia; *R* right

### Correlations results

As characterized in Fig. 3, negative correlation was found between the ASC and causal inflow from the left cuneus to the left PTH in MWCA vs. HC (*r* = −0.72, *P* = 0.004). Positive correlation was found between the ASC and causal inflow from the right MFG to the right PTH (*r* = 0.62, *P* = 0.019) in MWCA vs. HC.

## Discussion

The present rs-fMRI study with Granger causality analysis demonstrated effective connectivity alterations from and to the bilateral PTH in MWCA comparing to MWoCA and HC respectively. Specifically, the abnormal effective connectivity pathways included: 1) one thalamo-cortico-thalamic circuit and one ipsilateral feedback inflow to the PTH in MWCA compared with MWoCA, i.e., bidirectional connectivity between the bilateral PTH and the mPFC (including the ventral and dorsal part), and inflow from the left limbic regions (including the amygdala, hippocampus, and BG) to the ipsilateral PTH; 2) one bidirectional thalamic-cortical circuit and another two ipsilateral feedback inflows to the PTH in MWCA compared with HC, i.e., bidirectional connectivity between the right PTH and the bilateral temporoparietal regions (including the STG and IPL), inflows from the left parietooccipital regions (including the precuneus, cuneus, and PCC) to the ipsilateral PTH and from right DLPFC (including the IFG and MFG) to the ipsilateral PTH. Moreover, the disturbed effective connectivities between PTH and cuneus, as well as PTH and MFG were associated with the severity of allodynia. These results confirmed the previous notion that central sensitisation of the third-order trigeminal neurons in the thalamus was implicated in the mechanism of CA in migraine [14, 41]. Additionally, some regions in those abnormal effective connectivity pathways were involved in affective, cognitive, and sensory discriminative dimensions of pain, which suggested that CA implicated a multi-dimensional pain processing dysfunction.

One remarkable finding of the present study was the abnormally causal connectivity between the bilateral PTH and the mPFC (including the dorsal and ventral mPFC) in both MWCA vs. MWoCA and MWCA vs. HC. Results of the comparison between the two migraine groups suggested that CA may be associated with the decreased inflow from the left dmPFC to the ipsilateral PTH and increased inflow from the right vmPFC to the ipsilateral PTH. mPFC has widespread anatomical and functional connections with thalamus [42], and is thought to play a critical role in emotional-cognitive processing [43]. Furthermore, the two subregions of mPFC (i.e., vmPFC and dmPFC) have functional differentces,: the dmPFC is involved in evaluation of the perceived pain sensation [44], while the vmPFC has an important role in regulation of sensory and affective pain [45]. Therefore, the present results suggested that patients with CA had an impaired ability of evaluation of the perceived pain sensation. This was consistent with the definition of CA which is defined as pain (an exaggeration of the sensory inputs) resulting from the application of a non-noxious stimulus such as heat, cold or pressure to normal skin. Results also suggested that MWCA showed increased descending pain modulation which might be considered a response to the exaggerated pain information. Previous study has suggested that the medial thalamus and the mesial cortex (including the mPFC and ACC) involve the affective-motivational component of pain [46]. Other resting-state fMRI studies also found abnormal mPFC activity to be implicated in the abnormal affective pain perception, cognition, and modulation in migraineurs [25, 36, 47]. However, whether the dysfunctional effective connectivities between PTH and mPFC contribute to CA or are just cumulative effective results of CA still merits further study.

Another abnormal effective connectivity associated with CA was the decreased influence from the amygdala, hippocampus, putamen, and pallidum to the left PTH. All these subcortical regions have dense connections to thalamus [18, 48], and have been implicated in the cognitive/affective aspect of pain processing in migraine [49–52]. The involvement of these subcortical regions may correlate with the strongly relevant individual concern, like fears and anxiety [53] resulting from recurring and repetitive attacks of migraine [54]. Furthermore, the result is supported by a previous fMRI study which found that migraine patients with very high frequency of migraine attacks per month in response to the thermal stimuli demonstrated significantly lower activation throughout the caudate, putamen, and pallidum nuclei of the BG than those with low frequency of migraine attacks [51]. Therefore, based on these findings, we speculate that present abnormal cognitive and emotional pain processes may result from the accumulating damages from recurring and repetitive attacks, which may involve the cognitive-emotional impairment in migraineurs with CA.

Reduced influence from the left parietooccipital area (including cuneus, precuneus, and PCC) to the ipsilateral PTH was also found in MWCA comparing with HC, and was negatively correlated with the ASC (allodynia assessment index), which suggested the abnormal feedback flow through the parietooccipital-thalamic circuit to be implicated in the underlying mechanism of CA. Most of these regions are important parts of the default mode network (DMN), which is the most consistently reported intrinsic connectivity networks during resting-state and has been implicated in sensation integration, self-relevant, and internally cognitive-attentional dimensions of pain in migraineurs [55, 56]. Decreased connectivity between the parietooccipital regions and PTH reported herein may suggest an abnormal feedback from DMN regions to PTH, and certainly affects the following pain processing as relevant to the DMN function in migraine. In addition, the results also found increased influence from the right DLPFC (including IFG and MFG) to ipsilateral PTH in MWCA vs. HC, as well as its positive relationship with ASC. Concerning pain perception, the prefrontal regions including IFG have an important role in modulating pain processing during attentional manipulation paradigms [57]. DLPFC, along with medial thalamus, has also been reported to engage in the pain processing during heat allodynia [58]. Thus we speculate that the DLPFC exerts abnormal active control on sensory integration involving in the CA through the cortico-thalamus pathway.

The result revealed an abnormal bidirectional interaction between the right PTH and the bilateral temporoparietal areas (including the STG and IPL) in MWCA vs. HC. Specifically, there were increased inflow from the bilateral temporoparietal area to the right PTH and decreased outflow from the right PTH to the bilateral temporoparietal areas. Previous morphological and functional studies in migraine showed that migraineurs have reduced gray matter in STG and IPL [59, 60], as well as less pain-induced activation of STG than HC in the interictal state [61]. STG is an essential structure involved in auditory processing and social cognition [62], and is generally associated with the IPL to serve the so-called ventral stream of the visual pathway [63], which acts as a link between auditory and visual processing, perception and memory [64]. When taken together with the functional properties of these brain regions, the present findings suggested that migraineurs suffered from a condition of global dysfunction of sensory integration and memory processes, as well as abnormal descending modulation through the cortico-thalamic pathway.

### Study limitations

There are several limitations in the present study. First, the sample size of patients was small. The severity of allodynia symptoms, the length of disease course, the history of medication use and other characteristics were possible confounding variables, but we could not make any subgroup comparisons due to the few participants. Second, the current study did not present the large results of the MWoCA vs. HC, because we exclusively focused on the results that was directly associated with CA comparing MWCA with either MWoCA or HC. Finally, we have not performed a long-term evaluation or rescanned the subjects yet, so whether the functional alteration is a predictor or a consequence of central sensitization cannot be determined by the present study.

## Conclusions

The present study provides evidence of the involvement of the third-order trigeminovascular neurons in the posterior thalamic nuclei in the pathophysiological mechanism of CA in migraine. The disrupted effective connectivity pathways between PTH and other cortical or subcortical regions (such as the prefrontal cortex, limbic regions, parietooccipital and temporoparietal areas) exhibit a dysfunctional multi-dimensional (including the sensory-discriminative, cognitive, and affective domains) pain processing pattern in MWCA. These findings highlight the dysfunctional ascending and descending pain network at the thalamic-level. In a future study, the implicated regions in the present study will be chosen as seed regions for further effective connectivity analysis, in order to construct a comprehensive pathway model specifically accounting for CA in migraine.

## Abbreviations

ACC: anterior cingulate cortex
ASC: Allodynia symptom checklist
BG: basal ganglia
CA: cutaneous allodynia
DLPFC: dorsolateral prefrontal cortex
DMN: default mode network
dmPFC: dorsal medial prefrontal cortex
GCA: granger causality analysis
HC: healthy control
IFG: inferior frontal gyrus
IPL: inferior parietal lobule
MCC: middle cingulate cortex
MFG: middle frontal gyrus
mOFC: medial orbitofrontal cortex
mPFC: medial prefrontal cortex
mSFG: medial superior frontal cortex
MWCA: migraineurs with cutaneous allodynia
MWoCA: migraineurs without cutaneous allodynia
OC: olfactory cortex
PCC: posterior cingulate cortex
PTH: posterior thalamus
RG: rectus gyrus
rs-fMRI: resting-state functional magnetic resonance imaging
SMA: supplementary motor area
SMG: supramarginal gyrus
STG: superior temporal gyrus
vmPFC: ventral medial prefrontal cortex
